# The Effects of Combined Adiponectin-Metformin on Glucose and Lipids Levels in Mice and Acute Toxicity and Anti-Ulcerogenic Activity of Adiponectin Against Ethanol-Induced Gastric Mucosal Injuries in Rat

**DOI:** 10.3390/molecules16119534

**Published:** 2011-11-15

**Authors:** Atieh A. Fard, Maryam Hajrezaie, Farkaad A. Kadir, Fatemeh A. Sefideh, Suzy M. Salama, Zahra A. Al-Najar, Suhailah W. Qader, Mohammed A. Alshawsh, Mahmood A. Abdulla

**Affiliations:** 1 Institute of Biological Science, Faculty of Science, University of Malaya, 50603, Kuala Lumpur, Malaysia; Email: afard21@gmail.com (A.A.F.); maryam_hajrezaie@yahoo.com (M.H.); mahtab_ameri29@yahoo.com (F.A.S.); 2 Department of Anatomy, Faculty of Medicine, Cyberjaya University College of Medical Sciences, 63000 Cyberjaya, Selangor Darul Ehsan, Malaysia; Email: faalhadi@yahoo.com; 3 Department of Molecular Medicine, Faculty of Medicine, University of Malaya, 50603, Kuala Lumpur, Malaysia; Email: s.salama999@hotmail.com (S.M.S.); zahraa_alnajaar@yahoo.com (Z.A.A.-N.); alshaweshmam@yahoo.com (M.A.A.); 4 Department of Biological Science, Faculty of Biosciences and Bioengineering, University of Technology Malaysia, 81310, UTM Skudai, Johor, Malaysia; Email: suhaylaqadir@yahoo.com

**Keywords:** adiponectin, metformin, blood glucose, gastric ulcer, acute toxicity

## Abstract

Adiponectin is a protein hormone secreted entirely by abdominal fat tissue. It exhibits various biological activities. The present study was performed to evaluate the effects of metformin alone or in combination with adiponectin on blood glucose, TG (triglyceride), CHOL (Total cholesterol), LDL (Low density lipoprotein) and HDL (High density lipoprotein) levels in mice and also to evaluate the anti-ulcerogenic activity of adiponectin against ethanol induced gastric mucosal injury in rats. Three groups of mice were gavaged with 1% volume/body weight high fat-sucrose. Metformin at a dosage of 250 mg/kg was added to the feed and a dosage of 2.5 mg/kg adiponectin was injected intraperitoneally (i.p). Blood glucose was measured at one hour intervals for five hours. Blood concentrations of TG, CHOL, LDL and HDL were also measured at the end of the fifth hour of the experiment. On the other hand, four groups of adult healthy rats were i.p. injected with distilled water, omeprazole 20 mg/kg, 2.5 mg/kg and 5 mg/kg adiponectin one hour before oral administration of absolute ethanol to generate gastric mucosal injury. After an additional hour the rats were sacrificed and the ulcer areas of the gastric walls were determined. Furthermore, an acute toxicity study has indicated no mortality with 5 mg/kg dose of adiponectin injected i.p in rats and no major clinical signs of toxicity were observed. The results indicate that the effect of a combination of metformin and adiponectin on blood glucose and HDL is quite effective. Histology of the gastric wall of negative control rats revealed severe damage of gastric mucosa, along with edema and leucocyte infiltration of the submucosal layer compared to rats pre-treated with either omeprazole or adiponectin extract where there was marked gastric protection along with reduction or inhibition of edema and leucocytes infiltration. The results suggest that combination of metfomin and adiponectin give a promising antidiabetic effect and also, adiponectin promotes ulcer protection as ascertained by the comparative decrease of ulcer areas, reduction of edema and leucocytes infiltration of the submucosal layer.

## 1. Introduction

Improvements in technology lead to a sedentary lifestyle and consequently, an increase in body weight [1]. Obesity is associated with a high fat (HF) diet and this circumstance subsequently induces insulin resistance which is a major risk factor for diabetes and cardiovascular diseases [2]. Type 2 diabetes mellitus (T2D), which in earlier times was called non-insulin dependent diabetes, is nowadays one of the most common diseases. It is known that T2D is a metabolic disorder that is noticeable through increased levels of plasma glucose above the normal physiological range and is characterized by a progressive loss of glycemic control [3].

Adiponectin is one of the most important proteins which plays a basic role in insulin sensitivity, blood glucose and lipids. Studies leading to the knowledge of the exact mechanism and effects of this protein on blood glucose and lipid profile could lead to the reduction in risk of type 2 diabetes. Adiponectin is a protein which was originally identified in 1995 by Scherer [4]. It is encoded by the *AdipoQ* gene in humans, and is an adipokine that has recently attracted much attention [5,6]. Although adiponectin appears to exist as a full length protein in plasma, but it can also exist as smaller, globular fragments as reported by Lodish’s group [2,6]. It is assumed that adiponectin is totally secreted by the adipose tissue. Although its mechanisms of action, active forms, receptors and signaling pathways so far remain incompletely understood, some beneficial roles of this protein such as improved insulin sensitivity, glucose tolerance and lipid profile, have been identified [6]. It is interesting to note that [7] suggested that increased adiponectin levels might be associated with better inflammation reduction in diabetic subjects [7]. The risk factors including obesity and diabetes affect our arteries. Since cholesterol deposits in the arteries of patients with type 2 diabetes are much higher, an increase in adiponectin levels might be a helpful target for decreasing the atherosclerotic risk present in diabetic [7].

Due to the anti-inflammatory properties of adiponectin, the effects of this protein on other inflammatory conditions such as gastric ulcer, which is one of the most common gastrointestinal diseases that leads to treatment costs of up to millions of dollars annually, is worth studying. Gastric ulcers occur when the lining of organs is corroded by the acidic digestive juices which are secreted by the stomach cells. A number of agents are available for the treatment of diabetes [8] and gastric ulcer. Among them metformin is an oral diabetes medicine that is considered an insulin sensitizer, controlling blood sugar levels by decreasing the liver uptake of lactate. It belongs to the biguanide class (dimethylbiguanide) of anti-diabetic drugs and is the most widely prescribed medicine for the treatment of type 2 diabetes mellitus [9]. Metformin suppresses endogenous glucose production and also helps diabetic patients lose weight or, at least, maintain a stable weight [3,10]. It lowers glucose concentrations in diabetic patients by increasing glucose uptake and decreasing glucose production. Although the precise mechanisms of actions of the biguanides are not definitely understood, they are mostly attributed to an increase in nonessential tissue sensitivity to insulin, reduction in hepatic gluconeogenesis, and decrease in intestinal glucose absorption [3,9,10].

Since adiponectin plays an important role in the suppression of the metabolic disarrangement that may result in type 2 diabetes and the levels of adiponectin being reduced in diabetics compared to non-diabetics a combination of this protein with a common medicine such as metformin could be potentially beneficial as a treatment. In the present study, examination and characterization of recombinant adiponectin in *P. pastoris *was investigated*.* Expression of adiponectin was confirmed through SDS-PAGE and Western blotting. In addition, we used a rat model to investigate the antiulcerogenic activity of adiponectin against ethanol-induced gastric mucosal injuries and investigated of the effects of a combination of metformin and adiponectin on *in vivo* levels of blood glucose and blood lipids in mice.

## 2. Results and Discussion

### 2.1. Confirmation of Expression of Adiponectin through SDS-PAGE and Western Blotting

**Figure 1:**
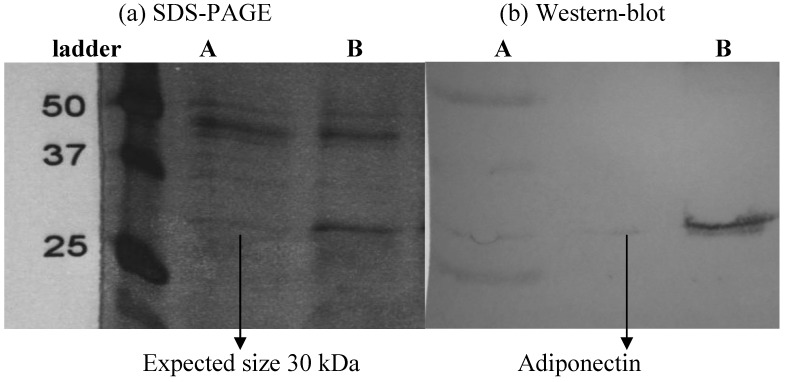
Confirmation of adiponectin expression (**a**) by SDS-PAGE: Presence of a band at 25-37 kDa; (**b**) and Western blotting.

In this study a SDS-PAGE analysis confirmed the expected 30 kDa size of the adiponectin protein (Figure 1a) and Western-blot confirmed the presence of the adiponectin protein. Western blotting showed that the expression band has strong reactivity against monoclonal mouse Anti-His antibody (Figure 1b), which indicated that recombinant protein has successfully been expressed in *P. pastoris* system.

### 2.2. Elaboration of the Glucose Level

**Figure 2:**
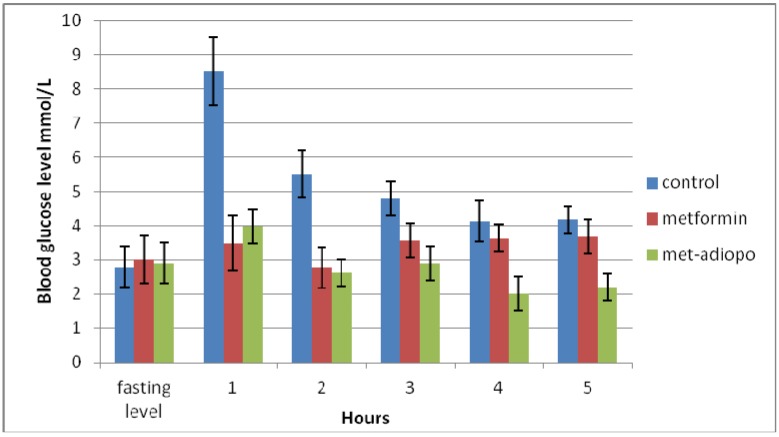
The blood glucose was just under 3 mmol/L at fasting level as calculated by glucometerbefore feeding the experimental mice. The graph shows a significant reduction of blood glucose level in the animals treated with metformin alone or in combination with adiponectin group at the 1st, 2nd, 3rd, 4th and 5th hours compared with control group.

Overall, there were significant reductions in glucose level in groups treated with metformin alone or in combination with adinopectin initially and at the 1st, 2nd, 3rd, 4th and 5th hours compared with the control group (Table 1). At 1st h post feeding there is significant reduction of glucose level in the metformin group compared with the metformin-adenopectin group. However, there were significant reductions of glucose levels at the 2nd, 3rd, 4th and 5th hours in the metformin-adenopectin group compared with the metformin group (Table 1) and (Figure 2).

**Table 1:** Blood glucose levels.

	Fasting level *	1 h	2 h	3 h	4 h	5 h
Control	2.80 + 0.06	8.25 + 0.01 ^a^	5.51 + 0.01 ^a^	4.78 + 0.01 ^a^	4.13 + 0.03 ^a^	4.17 + 0.01 ^a^
Metformin	3.00 + 0.06	3.48 + 0.01 ^b^	2.78 + 0.01 ^b^	3.56 + 0.01 ^b^	3.63 + 0.01 ^b^	3.68 + 0.02 ^b^
Metformin-adiponectin	2.90 + 0.06	3.98 + 0.01 ^c^	2.62 + 0.01 ^c^	2.9 + 0.01 ^c^	2.00 + 0.04 ^c^	2.20 + 0.06 ^c^

All values are expressed as mean ± SEM. * units = mmol/L. Means with different superscripts are significantly different at the 0.05 level.

### 2.3. Measurement of LDL and HDL in Blood

There were no significant differences in HDL levels between groups after treatment (Table 2). However, there were significant increases in the LDL level in metformin group compared with control group or the metformin-adenopectin group (Table 2).

**Table 2:** Total Cholesterol,Triglyceride,HDL and LDL levels after treatment.

Groups	Triglyceride *	Cholesterol *	HDL *	LDL *
Control	0.8 ± 0.03 ^a^	2.00 ± 0.06 ^b^	0.91 ± 0.004	0.47 ± 0.01 ^b^
Metformin	0.42 ± 0.01 ^b^	2.20 ± 0.06 ^a^	0.92 ± 0.01	0.53 ± 0.01 ^a^
Metformin-adiponectin	0.46 ± 0.01 ^b^	1.90 ± 0.03 ^b^	0.92 ± 0.01	0.45 ± 0.01 ^b^

All values are expressed as mean ± SEM. * units = mmol/L. Means with different superscripts are significantly different at the 0.05 level.

### 2.4. Measurement of Total Cholesterol & Triglyceride Level in Blood

There were significant reductions of triglyceride levels in the metformin group or the metformin-adenopectin group compared with control group (Table 2). However, there were no significant differences in triglyceride levels between the metformin group and the metformin-adenopectin group (Table 2). Cholesterol levele were significantly increased in the metformin treated group compared with metformin-adenopectin group or control group (Table 2), but there were no significant differences in cholesterol levels between control group and metformin-adenopectin group (Table 2).

### 2.5. Acute Toxicity Study

An acute toxicity study was carried out in which rats injected i.p. with the adiponectin at a dose of 2 and 5 mg/kg of adiponectin were kept under observation for 14 days. All the animals remained alive and did not manifest any significant signs of toxicity. There were no abnormal signs, behavioral changes, body weight changes, or macroscopic findings at any time of observation. There was no mortality at the above-mentioned doses at the end of 14 days of observation. Histological examination of liver and kidney, hematology and serum biochemistry revealed no significant changes between the different groups. From these results it is concluded that the adiponectin is quite safe, even at these higher doses and has no acute toxicity and the oral lethal dose (LD_50_) for the male and female mice was greater than 5 mg/kg body weight.

#### 2.5.1. Behavioural Observation and Mortality

The i.p. injection of adiponectin did not cause mortality among any of the groups (Table 3). In addition, no behavioural signs of toxicity were observed throughout the period of the study period among all groups.

**Table 3:** The observation data for toxicology study of adiponectin.

Dose	Occurrence of mortality				
	30 min	2 h	4 h	24 h	48 h
5 mg/kg	0/6	0/6	0/6	0/6	0/6
2 mg/kg	0/6	0/6	0/6	0/6	0/6
Vehicle *	0/6	0/6	0/6	0/6	0/6

* Distilled water.

#### 2.5.2. Haematology

Total white blood cells were counted for all rats. Table 4 shows that there are no significant differences among any of the groups.

**Table 4:** Total white blood cell (WBC count (10^9^/L).

Group	Male	Female
Vehicle (distilled water)	8.45 ± 1.76	8.28 ± 2.3
Adiponectin (2 mg/kg)	7.75 ± 1.95	7.71 ± 2.21
Adiponectin (5 mg/kg)	8.05 ± 1.62	7.48 ± 1.34

All values expressed as mean ± S.E.M. There were no significant differences among groups.

#### 2.5.3. Serum Biochemistry

The parameters of liver functions that have been tested were aspartate aminotransferase (AST), alanine aminotransferase (ALT), total protein (TP), albumin, globulin, total bilirubin (TB), conjugated bilirubin (CB), alkaline phosphatise (AP), gamma-glutamyl transferase (GGT). Their levels were analyzed as indication of liver functions compared to their vehicle group. Furthermore, the level of urea, creatinine, anion gap and serum electrolytes (CO_2_, potassium, sodium and chloride) of all groups were determined as markers of kidneys functions (Table 5 and Table 6).

**Table 5:** Effect of adiponectin on renal function test in acute toxicity study.

Dose	Sodium (mmol/L)	Pottasium (mmol/L)	Chloride (mmol/L)	CO_2_ (mmol/L)	Anion gap (mmol/L)	Urea (mmol/L)	Creatinine (µmol/L)
Vehicle (Distilled water)	138.335 ± 1.46	5.15 ± 0.19	103.05 ± 1.17	22.11 ± 0.81	18.13 ± 0.76	5.16 ± 0.43	50.05 ± 1.32
Low Dose (2 mg/kg)	137.85 ± 1.44	5.05 ± 0.16	102.49 ± 1.21	21.94 ± 0.17	18.09 ± 1.03	4.98 ± 0.41	48.93 ± 0.81
High Dose (5 g/kg)	138.04 ± 1.52	4.98 ± 0.15	103.76 ± 1.76	22.04 ± 0.84	17.95 ± 0.57	5.33 ± 0.38	49.70 ± 1.80

All values expressed as mean ± S.E.M. There are no significant differences between groups. Significant value at p < 0.05.

**Table 6:** Effect of adiponectin on liver function test in acute toxicity study.

Dose	Total protein (g/L)	Albumin (g/L)	Globulin (g/L)	TB (µmol/L)	CB (µmol/L)	AP (IU/L)	ALT (IU/L)	AST (IU/L)	GGT (IU/L)
Vehicle	71.24 ± 1.41	11.28 ± 0.75	60.11 ± 1.63	1.97 ± 1.24	0.95 ± 1.13	134.78 ± 5.36	53.18 ± 3.61	153.86 ± 8.22	4.96 ± 0.96
Low Dose(2 mg/kg)	71.31 ± 1.33	11.53 ± 0.64	59.73 ± 1.45	2.15 ± 1.17	0.97 ± 1.17	133.63 ± 4.54	52.94 ± 2.87	154.14 ± 5.61	5.02 ± 1.35
High Dose(5 mg/kg)	70.81 ± 1.13	11.49 ± 0.47	60.07 ± 1.17	1.91 ± 1.09	0.92 ± 1.33	134.25 ± 4.22	52.87 ± 3.15	155.02 ± 5.13	5.32 ± 1.48

All values expressed as mean ± S.E.M. There are no significant differences between groups. Significant value at p < 0.05; TB: Total bilirubin; CB: Conjugated bilirubin; AP: Alkaline phosphatase; ALT: Alanine aminotransferase; AST: Aspartate aminotransferase; GGT: G-Glutamyl Transferase. Vehicle = distilled water.

#### 2.5.4. Gross Necropsy and Histology

**Figure 3:**
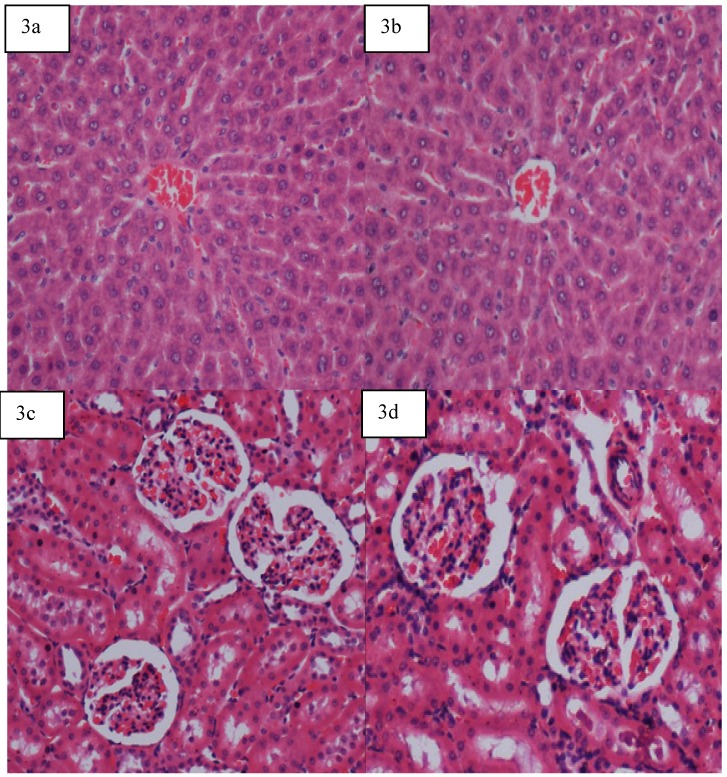
Histology and pathology snapshots of (**3a**) liver tissue in a rat pre-treated with vehicle (distilled water) showed normal appearance, (**3b**)liver tissue in a rat pre-treated with 5 mg/kg adiponectin showed normal appearance(**3c**) kidney tissue in a rat pre-treated with vehicle (distilled water) showed normal appearance (**3d**) kidney tissue in a rat pre-treated with 5 mg/kg adiponectin showed normal structural appearance.

There was no significant decrease or increase in levels of all parameters of males as well as females groups in all dosages, as shown in Figure 3(a) to 3(d).

### 2.6. Anti-Ulcer Activity

#### 2.6.1. pH of Gastric Content and Mucus Production

The effect of adiponectin on gastric acidity in the ethanol-induced gastric lesion model is reported in Table 8. The acidity of gastric content in experimental animals pretreated with adiponectin or omeprazole was significantly decreased compared with that of the ulcer control group (p < 0.05). Also the gastric acidity was significantly decreased in the omeprazole treated group compared with the adenopectin groups. However, there were no significant differences between adenopection groups. The mucus production of gastric mucosa were significantly increased (p < 0.05) in animals pretreated with adiponectin or omeprazole compared with ulcer control group (Table 7). However, there were no significant differences in mucus content between omeprazole and adenopectin groups.

**Table 7:** Effect of adenopectin on pH of gastric content and mucus in rats.

Animal Group	Pre-treatment(i.p.) injection	pH of gastric content (Mean ± S.E.M)	Mucus content
1	Distilled water (Ulcer control)	3.34 ± 0.01 ^c^	0.36 + 0.01 ^b^
2	Omeprazole (20 mg/kg)	6.71 ± 0.33 ^a^	0.55 + 0.02 ^a^
3	Adiponectin (2.5 mg/kg)	4.79 ± 0.01 ^b^	0.52 + 0.01 ^a^
4	Adiponectin (5 mg/kg)	5.01 ± 0.01 ^b^	0.54 + 0.02 ^a^

All values are expressed as mean ± standard error mean. Means with different superscripts are significantly different. The mean difference is significant at the 0.05 level.

#### 2.6.2. Gross Evaluation of Gastric Lesions

**Figure 4:**
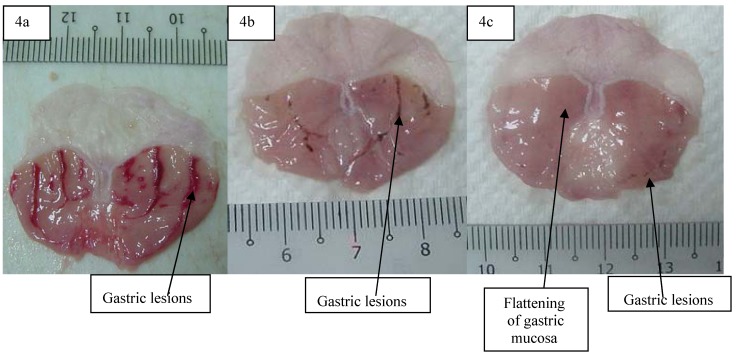
Gross appearance of the gastric mucosa in (4a) a rat pre-treated with distilled water (negative control). Severe injuries are seen (4b) in the gastric mucosaof a rat pre-treated with omeprazole (20 mg/kg). Injuries to the gastric mucosa are milder compared to the injuries seen in the negative control rat(4c) a rat pre-treated with 5 mg/kg of adiponectin. Mild injuries to the gastric mucosa are seen, and flattening of gastric mucosa is shown.

The anti-ulcer activity of adiponectin in the ethanol-induced gastric lesion model is shown in Table 8. Results showed that rats pre-treated with adiponectin before being given absolute ethanol had significantly reduced areas of gastric ulcer formation compared to rats pre-treated with only distilled water (ulcer control group) (Figure 4 a, b and c) which means to say that the adiponectin significantly suppressed the formation of ulcers. Moreover it was interesting to note the flattening of gastric mucosal folds in rats pre-treated with adiponectin. It was also observed that protection of gastric mucosa was more prominent in rats pre-treated with 5 mg/kg adiponectin (Table 8). Furthermore, ethanol-induced mucosal damage was significant and dose dependently reduced in the size and severity by pre-treatment of the animals with adiponectin. The significant inhibition of gastric ulcer after pre-treatment with adiponectin by dissolving it with distilled water and i.p. administration to rats in concentrations of 20 mg/kg body weight was comparable with omeprazole, which is a standard drug used for curing gastric ulcers [11]. Omeprazole is a proton pump inhibitor which has been widely used as an acid inhibitor agents for the treatment of disorders related to gastric acid secretion for about 15 years [12]. Omeprazole is a substituted benzimidazole that inhibits acid secretion by acting on the hydrogen-potassium exchanger (H+, K+-ATPase) for the apical plasma membrane of the gastric mucosa [13].

**Table 8:** Observed ulcer area and inhibition percentage in rats.

Animal Group	Pre-treatment (i.p.) injection	Ulcer area (mm)^2^ (mean ± S.E.M)	Inhibition (%)
1	Distilled water (Ulcer control)	825.33 ± 11.86 ^a^	-
2	Omeprazole (20 mg/kg)	183.00 ± 8.47 ^b^	57.79%
3	Adiponectin (2.5 mg/kg)	201.17 ± 9.00 ^c^	75.63%
4	Adiponectin (5 mg/kg)	97.33 ± 8.02 ^d^	88.23%

All values are expressed as mean ± standard error mean. Means with different superscripts are significantly different. The mean difference is significant at the 0.05 level.

Omeprazole is highly selective for the proton pump and undergoes a catalyzed conversion into active form within the acid forming space. The active inhibitors react with SH (thiol) groups of the proton pump, resulting in inhibition of acid formation. Omeprazole blocks the enzymes in the wall of the stomach from producing acid. A decrease in production of stomach acid would thus allow the stomach to heal [14].

#### 2.6.3. Histological Evaluation of Gastric Lesions

**Figure 5:**
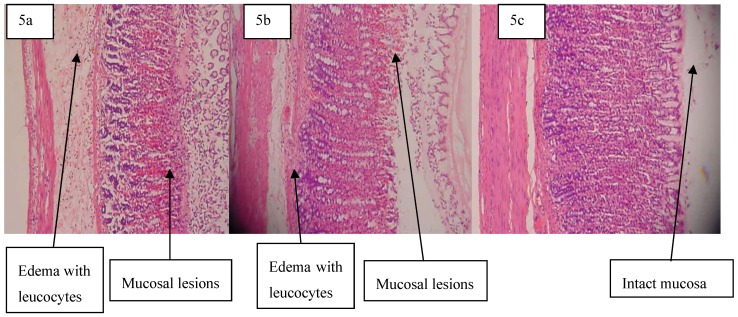
Histology and pathology snapshots of gastric mucosa (**5a**) in a rat pre-treated with distilled water only. There is severe disruption to the surface epithelium, and edema of the submucosa layer with leucocyte infiltration,(**5b**)in a rat pre-treated with omeprazole (20 mg/kg). There is mild disruption to the surface epithelium with mild edema and leucocyte infiltration of the submucosal layer, (**5c**) in a rat pre-treated with 5 mg/kg of adiponectin. There is mild disruption to the surface epithelium with no edema and no leucocyte infiltration of the submucosal layer.

Histological observation of ethanol-induced gastric lesions in the ulcer control group pre-treated with distilled water only showed comparatively extensive damage to the gastric mucosa, edema and leukocyte infiltration of the submucosal layer (Figure 5a). Rats that received pre-treatment with adiponectin had comparatively better protection of gastric mucosa as seen by the reduction in ulcer area, reduced or absent of sub mucosal edema and leukocyte infiltration. (Figure 5b and Figure 5c). Adiponectin has thus been shown to exert its cytoprotective effects in a dose-dependent manner.

One of the adipocyte-specific plasma proteins which are secreted by adipose tissue like other adipocytokines, is adiponectin. Adiponectin modulates a variety of metabolic processes, including glucose regulation and fatty acid catabolism [15]. Adiponectin displays a multiplicity of functions, such as antiatherogenic and antidiabetic properties [16], and also acts as an endogenous regulator of endothelial cells in response to inflammatory stimuli and regulates the metabolism of lipids and proteins [16,17]. Although adiponectin is secreted from adipose tissue, it is well known that low levels of this protein are present in obese people, which means the levels of this hormone are inversely correlated with body fat percentage in adults [2,10,18]. Adiponectin is secreted into the bloodstream and it accounts for 0.01% of total plasma protein. Plasma concentrations of adiponectin reveal a sexual dimorphism with males having lower levels than females [16,19]. A number of oligomeric forms of adiponectin have been described in blood [20]. Initially three adiponectin molecules bind together to form a homotrimer. Like the plasma concentration, the relative levels of the higher-order structures are sexually dimorphic, where females have increased proportions of the high-molecular weight forms [21].

Production of adiponectin thus is very important for basic research and clinical applications. According to preparation for the operation and production systems compared to recent studies, one of the increasingly popular cellular hosts for expressing the adiponectin is the methylotrophic yeast, *P. pastoris* [22]. The *P. pastoris* expression systems have been successfully developed for the production of a variety of heterologous recombinant proteins [23,24].

High triglyceride levels and decreased HDL cholesterol levels, but relatively small differences in LDL are the most common patterns of dyslipidemia in type 2 diabetics [25]. The mean triglyceride level in type 2 diabetes is <200 mg/dL and 85-95% of patients have triglyceride levels below 400 mg/dL. In diabetics, optimal LDL cholesterol levels are <100 mg/dL, optimal HDL cholesterol levels are >45 mg/dL and optimal triglyceride levels are <200 mg/dL. The study of [26] indicates that metformin is one of the anti-diabetic drugs which has opposing effects on adiponectin protein expression and releases in differentiated adipocytes.

Since metformin is a potent insulin-sensitizing agent that acts primarily on hepatic glucose production and has additional effects on peripheral insulin sensitivity, results from the present study indicate that the effects of a combination of metformin and adiponectin on blood glucose are more effective than the effects of adiponectin itself. The study of adiponectin by [27] showed that the values of fasting and random blood glucose, serum triglycerides and LDL cholesterol were significantly increased in diabetic patients as compared with normal control subjects, except for serum adiponectin and HDL cholesterol concentrations which were significantly decreased in diabetic patients [27]. On the other hand, regarding to [21] the pharmacological effect of adiponectin on dropping insulin resistance is correlated to a decline in triglyceride content and in plasma fatty acid levels in liver and muscle.

Although metformin increases lipolysis and reduces triglyceride stores in adipocytes [26], and triglyceride accumulation is significantly reduced by supporting the oxidation of lipid treatment of diabetic animals with adiponectin, our study shows that there is no significant difference in the combination of adiponectin and metformin on triglycerides, compared with metformin alone [28]. This may be because of suppression of adiponectin production through metformin action in different adipocytes [26].

Since cholesterol levels are closely associated with LDL levels, when total cholesterol levels increased, LDL would do so too. Schmitt *et al.* suggested that LDL uptake by fibroblasts may be impaired in type 2 diabetes. This leads to an increase in LDL: HDL ratio in type 2 diabetics. In our study, the LDL ratio did not differ significantly between the control and metformin-adiponectin groups (*p* value >0.05). No significant difference was found in the total cholesterol levels in the metformin- adiponectin group compared to other groups of mice [29].

Adiponectin has also been proven by recent studies to be a remarkable protective factor against development of atherosclerosis due to its anti-inflammatory effects. Now by this theory it is believed that the use of recombinant adiponectin is helpful to avoid cardiovascular disease [21]. It is known that gastric lesions produced by ethanol administration appear as multiple-hemorrhagic red bands of different size along the glandular stomach. Absolute ethanol is commonly used for inducing ulcers in experimental rats and leads to intense gastric mucosal damage. In the present study, flattening of the mucosal folds was observed, which suggests that gastro-protective effect of adiponectin might be due to a decrease in gastric motility. It is reported that the changes in the gastric motility may play a role in the development and prevention of experimental gastric lesions [30]. Relaxation of circular muscles may protect the gastric mucosa through flattening of the folds. This will increase the mucosal area exposed to necrotizing agents and reduce the volume of the gastric irritants on the rugal crest [30,31]. Ethanol produces a marked contraction of the circular muscles of rat fundic strip. Such a contraction can lead to mucosal compression at the site of the greatest mechanical stress, at the crests of mucosal folds leading to necrosis and ulceration [30].

The result of the present study also revealed protection of gastric mucosa and inhibition of leucocyte infiltration of gastric wall in rats pre-treated with adiponectin. Similarly, [30] demonstrated that the reduction of neutrophil infiltration into ulcerated gastric tissue promotes the prevention of gastric ulcers in rats. This study evaluated the activity of adiponectin on gastric ulcer compared to omeprazole for the first time. In the current study, the rats pre-treated with adiponectin have significantly reduced areas of gastric ulcer formation before being given absolute alcohol compared to rats pre-treated with only distilled water. This proves that the adiponectin significantly suppressed the creation of ulcers. Moreover, the present study indicated that the protection of gastric mucosa is more prevalent in rats pre-treated with adiponectin. Besides, ethanol-induced mucosal damage is significant and does dependently reduce in size and severity by pre-treatment of the animals with adiponectin. The significant inhibition of gastric ulcer in pre-treatment with adiponectin was compared with omeprazole which is a standard drug used for curing gastric ulcer. In histological observation, the rats that received pre-treatment with adiponectin had comparatively better protection of the gastric mucosa. Subsequently, adiponectin has been shown to exert cytoprotective effects in a dose-dependent manner.

To determine the safety of adiponectin for human use, toxicological evaluation is carried out in various experimental animals to predict toxicity and to provide guidelines for selecting a ‘safe’ dose in humans. Liver and kidney of the treated rats showed no significant change as compared to the control group. Clinical biochemistry values were within the range of the control animals tested and similar to some of the control reference values published elsewhere [30,32]. The highest dose of adiponectinwhich did not cause any toxicity was 5 mg/kg body weight, suggesting that adiponectin is relatively non-toxic since in acute toxicity studies, the product is considered non-toxic if no deaths are registered after 14 days of observation and no clinical signs of toxicity are observed at doses at or below 5 g/kg [30,33].

## 3. Experimental

### 3.1. Recombinant Yeast

Recombinant yeast was obtained from the adiponectin expression project which had been previously carried out [15]. Plasmid DNA was first linearized by the restriction enzyme *Sac I*, (Fermentas, Canada) and then transformed in to *P. pastoris *stain X33 (Prondisa, Canada) by using Easy Comp transformation kit (Invitrogen, USA), following the manufacturer’s instructions.

### 3.2. Growth Media Preparation for Protein Expression

Recombinant yeast was cultured in order to produce adiponectin. First, Buffered glycerol-complex (BMGY) media was used as a growth media having glycerol as carbon source. A single recombinant *P. pastoris* colony was inoculated into a BMGY medium and cells were harvested by centrifugation and pellets were mixed with buffered methanol-complex medium (BMMY). In order to separate the protein from the yeast, the culture medium was centrifuged and the supernatant was stored separately overnight at 20 °C. Acetone was added in order to precipitate the protein, and then kept at 20 °C overnight. After centrifuged the mixture, pellets were mixed with PBS potassium phosphate saline buffer which helped to separate soluble protein from insoluble protein. The solution was centrifuged and the supernatant contained the soluble protein was collected and subjected to purification process.

### 3.3. Purification of Adiponectin

Purification was done by nickel-based purification columns in order to obtain a sufficient quantity of purified adiponectin for subsequent use. Sodium phosphate buffer was applied to the columns and the process was followed by applying the supernatant to the columns. After that, binding buffer was applied to the columns and proteins containing His tags were bound to the nickel, whereas non-specific proteins were eluted from the column. The His tagged proteins were eluted with elution buffer that contained imidazole which displaced the His from the nickel. The eluant was collected into tubes.

### 3.4. Protein Study

#### SDS-PAGE-Western Blotting

In order to confirm the presence and estimated size of the adiponectin protein SDS-PAGE and Western blotting were performed.

### 3.5. Mice Preparation for Adiponectin-Metformin Combination

Eighteen healthy female ICR mice were used as a model system. Animals were obtained from the Animal House, Faculty of Medicine, University of Malaya in Kuala Lumpur (Ethics No. PM 07/05/2010 MAA (a) (R) to compare the biological activity of the adiponectin-metformin combination. Animals, each with mean body weight of 27 g were assigned equally into three groups labeled as: control, metformin and adiponectin-metformin groups. Following overnight fasting, the animals were gavaged with a 1% volume/body weight high fat-sucrose diet (12 mL water, 6 g sunflower oil, 6 g butter, 10 g sucrose and 10 g skim milk). Group 1 rats were given standard palate food and injected i.p. with 2.5 mL of distilled water. A dosage of 250 mg/kg/body weight metformin was given orally by adding to the feed of group 2 and 3 rats, and also Group 2 rats were injected i.p. with 2.5 mL distilled water. Adiponectin dosage of 2.5 mg/kg was injected intraperitoneally to group 3 rats.

Blood glucose was measured with a glucometer (Bayar Contour^™^, Hong Kong) at one hour intervals for 5 h. Available commercial kits were used to determine blood concentration of triglyceride (TG), total cholesterol (CHOL) (Siemens, U.S.A.), low density lipoprotein (LDL) and high density lipoprotein (HDL) (Dade Behring, U.S.A.).

### 3.6. Acute Toxicity Test and Experimental Animals

Adult healthy Sprague Dawley rats (6-8 weeks old) were obtained from the Animal House, Faculty of Medicine, University of Malaya, Kuala Lumpur (Ethics No. PM 27/07/2010 MAA (a) (R). The rats weighed between 180-200 g. The animals were given standard rat pellets and tap water *ad libitum*. The acute toxic study was used to determine a safe dose for the adiponectin. Thirty six rats (18 males and 18 females) were assigned equally into three groups labeled as vehicle (distilled water), Group 1, 2 mg/kg of adiponectin, Group 2 and 5 mg/kg of adiponectin Group 3. The animals were fasted overnight of food but not water, prior to dosing. Food was withheld for a further 3 to 4 h after dosing. The animals were observed after intraperitoneal injection (i.p.) of distilled water, 2 mg/kg and 5 mg/kg of adiponectin, respectively at 30 min and 2, 4, 8, 24 and 48 h for the onset of clinical or toxicological symptoms. Mortality, if any, was observed over a period of two weeks. No lethality was observed. The animals were fasted on the 14th day and sacrificed on the 15th. Histology, hematological and serum biochemical parameters were determined following standard methods [11,12,13,14,15,16,17,18,19,20,21,22,23,24,25,26,27,28,29,30,31,32,33,34]. Haematological (for total white blood cell count), serum biochemical parameters as follows: aspartate aminotransferase (AST), alanine aminotransferase (ALT), total protein, albumin, globulin, total bilirubin, conjugated bilirubin, alkaline phosphatase, gamma glutamyl transferase (GGT), urea, creatinine, anion gap and serum electrolytes (CO_2_, potassium, sodium and chloride) were determined. Gross necropsy and histopathology (at scheduled termination, all surviving animals were anaesthesia by overdose of xylazin and ketamine and quickly sacrificed by exsanguination of the jugular vein for blood sample collection. Gross post-mortem examinations were performed on all terminated animals [34]. Liver and kidney from each animal were routinely processed and embedded in paraffin. After sectioning and staining with the haematoxylin and eosin (H&E) stain method, all slides were observed under microscope with magnifications of ×10, ×20, ×40 and ×100 in order to observe any pathological changes) [30]. The study was approved by the Ethics Committee for Animal Experimentation, Faculty of Medicine, University of Malaya, Kuala Lumpur. All animals received human care according to the criteria outlined in the “Guide for the Care and Use of Laboratory Animals” prepared by the National Academy of Sciences and published by the National Institutes of Health.

### 3.7. Antiulcer Activity

#### 3.7.1. Experimental Animals

Sprague Dawley healthy adult male rats were obtained from the Experimental Animal House, Faculty of Medicine, University of Malaya, Kuala Lumpur (Ethic No. PM/27/07/2010/MA (A) (R)). The rats were divided randomly into four groups of six rats each. Each rat that weighted between 200-250 g was placed individually in a separate cage (one rat per cage) with wide-mesh wire bottoms to prevent coprophagia during the experiment. The animals were maintained on a standard pellet and tap water diet.

#### 3.7.2. Gastric Ulcer Induction by Ethanol

The rats fasted for 24 h before the experiment [11], but were allowed free access to drinking water up to 2 h before the experiment. Gastric ulcer was induced by orogastric intubation of absolute ethanol (5 mL/kg) according to the method described by [11]. The ulcer control groups were i.p. injected with vehicle (distilled water, 5 mL/kg). The reference group received i.p. injection with 20 mg/kg omeprazole in vehicle (5 mL/kg) as positive controls. The experimental groups were i.p. injected with 2.5 mg/kg and 5 mg/kg of adiponectin, respectively. One hour after this pre-treatment, all groups of rats were gavaged with absolute ethanol (5 mL/kg) in order to induce gastric ulcers [31]. The rats were euthanized by cervical dislocation 60 minutes later [11] under overdose of xylazin and ketamine anesthesia and their stomachs were immediately excised.

#### 3.7.3. Measurement of Acid in Gastric Juice

Each stomach was opened along the greater curvature. Samples of gastric contents were analyzed for hydrogen ion concentration by pH-meter titration with 0.1 N NaOH solutions using a digital pH meter. The acid content was expressed as mEq/L [11,31].

#### 3.7.4. Measurement of Mucus Production

Gastric mucus production was measured in the rats that were subjected to absolute ethanol-induced gastric mucosal injury. The gastric mucosa of each rat was gently scraped using a glass slide and the mucus obtained was weighed using a precision electronic balance [35].

#### 3.7.5. Gross Gastric Lesions Evaluation

Ulcers of gastric mucosa, appear as elongated bands of hemorrhagic lesions parallel to the long axis of the stomach. Gastric mucosa of each rat was thus examined for damage. The length and width of the ulcer on the gastric mucosa were measured by a planimeter (10 × 10 mm^2^ = ulcer area) under dissecting microscope (1.8×). The ulcerated area was measured by counting the number of small squares, 2 mm × 2 mm, covering the length and width of each ulcer band. The sum of the areas of all lesions for each stomach was applied in the calculation of the ulcer area (UA) wherein the sum of small squares × 4 × 1.8 = UA mm^2^ as described elsewhere [36]. The inhibition percentage (1%) was calculated by the following formula as described elsewhere [11]:

(I%) = [(UA_control_ − UA_treated_) ÷ UA_control_] × 100

#### 3.7.6. Histological Evaluation of Gastric Lesions

Specimens of the gastric walls from each rat were fixed in 10% buffered formalin and processed in a paraffin tissue processing machine. After embedding sections of the stomach were made at a thickness of 5 µm and stained with haematoxylin and eosin for histological evaluation [37].

### 3.8. Statistical Analysis

All values were reported as mean ± S.E.M. The statistical significance of differences between groups was assessed using one-way ANOVA. A value of p < 0.05 was considered significant.

## 4. Conclusions

Our study supports the hypothesis that a combination of adiponectin with metformin might have better effects on blood glucose levels and HDL. Moreover, our data suggests that the protein adiponectin plays a significant role on inflammatory diseases such as gastric ulcer, significantly suppressing ulcer formation. The acute toxicity profile of adiponectin could be considered favorable, judging from the absence of adverse clinical manifestations in experimental animals after 14 days of observation. It is concluded that adiponectin is quite safe, even at these higher doses, and has no acute toxicity and the oral lethal dose (LD_50_) for the male and female rats were greater than 5 mg/kg body weight.
